# Biopsia de tumores pulmonares con guía ecográfica: evaluación de eficacia y complicaciones

**DOI:** 10.31053/1853.0605.v80.n4.40922

**Published:** 2023-12-26

**Authors:** Juan Bautista Del Valle, Marina Alonso Serena, Gabriel Ducrey, Jésica Lorena Savluk, Matías Adrián Borensztein

**Affiliations:** 1 Hospital Italiano de Buenos Aires. Servicio de Diagnóstico por Imágenes Argentina

**Keywords:** biopsia guiada por imagen, biopsia, neoplasias pulmonares, ultrasonografía, image guided biopsy, biopsy, lung neoplasms, ultrasonography, biópsia guiada por imagem, biópsia, neoplasias pulmonares, ultrassonografia

## Abstract

**Introducción:**

El diagnóstico de cáncer pulmonar al igual que el de los nódulos pulmonares se encuentra en aumento. La biopsia percutánea se ha convertido en una herramienta trascendental para su diagnóstico. Tradicionalmente la tomografía computada es empleada para estos procedimientos por su capacidad para demostrar con claridad los huesos y el pulmón aireado. Sin embargo, en casos seleccionados puede efectuarse con ecografía.

**Métodos:**

Estudio retrospectivo realizado entre enero de 2020 y diciembre de 2021, durante la pandemia por SARS-CoV-2. Todos los pacientes tenían lesiones pulmonares de base pleural o lesiones pleurales, algunos con antecedentes conocidos de cáncer.

**Resultados:**

Se realizaron 36 procedimientos, en 32 (88,9%) la muestra obtenida presentó rédito diagnóstico y la prueba adicional más utilizada fue la Inmunohistoquímica en 23 (63,9%). Se reportaron complicaciones en 5 pacientes (13,9%): 2 con neumotórax leve, 2 con hemotórax (1 leve y 1 moderado) y 1 paciente refirió dolor.

**Conclusión:**

La ecografía es un método válido para ser usado como guía de biopsias de lesiones pleurales y pulmonares periféricas. La tasa de complicaciones y reedito diagnóstico ha demostrado estar en línea con la experiencia de otros autores y guías internacionales.

## Abstract (en)

**Introduction:**

The diagnosis of lung cancer, as well as that of lung nodules, is increasing. Percutaneous biopsy has become a transcendental tool for its diagnosis. Traditionally, computed tomography is used for these procedures because of its ability to clearly demonstrate bone and aerated lung. However, in selected cases it can be performed with ultrasound.

**Methods:**

Retrospective study conducted between January 2020 and December 2021, during the SARS-CoV-2 pandemic. All patients had pleural-based lung lesions or pleural lesions, some with a known history of cancer.

**Results:**

Thirty-six procedures were performed, in 32 (88.9%) the sample obtained presented diagnostic yield and the most used additional test was Immunohistochemistry in 23 (63.9%). Complications were reported in 5 patients (13.9%): 2 with mild pneumothorax, 2 with hemothorax (1 mild and 1 moderate) and 1 patient reported pain.

**Conclusion:**

Ultrasound is a valid method to be used as a guide for biopsies of pleural and peripheral pulmonary lesions. The complications and diagnostic rate has been shown to be in line with the experience of other authors and international guidelines.

## Resumo

**Introdução:**

O diagnóstico de câncer de pulmão, bem como de nódulos pulmonares, está aumentando. A biópsia percutânea tornou-se uma ferramenta transcendental para o seu diagnóstico. Tradicionalmente, a tomografia computadorizada é usada para esses procedimentos devido à sua capacidade de demonstrar claramente o osso e o pulmão aerado. Porém, em casos selecionados pode ser realizada com ultrassom.

**Métodos:**

Estudo retrospectivo realizado entre janeiro de 2020 e dezembro de 2021, durante a pandemia de SARS-CoV-2. Todos os pacientes apresentavam lesões pulmonares de base pleural ou lesões pleurais, alguns com história conhecida de câncer.

**Resultados:**

Foram realizados 36 procedimentos, em 32 (88,9%) a amostra obtida apresentou rendimento diagnóstico e o exame complementar mais utilizado foi a imuno-histoquímica em 23 (63,9%). Complicações foram relatadas em 5 pacientes (13,9%): 2 com pneumotórax leve, 2 com hemotórax (1 leve e 1 moderado) e 1 paciente relatou dor.

**Conclusão:**

A ultrassonografia é um método válido para ser usado como guia para biópsias de lesões pleurais e pulmonares periféricas. A taxa de complicações e reemissões diagnósticas tem se mostrado compatível com a experiência de outros autores e diretrizes internacionais.

**CONCEPTOS CLAVE**

**Qué se sabe sobre el tema**

La biopsia de lesiones pulmonares es una herramienta clave en el diagnóstico y estadificación del paciente oncológico. Sus indicaciones van más allá del espectro de las neoplasias: es posible realizar análisis de laboratorio para diagnóstico de procesos infecciosos. Estos procedimientos, en forma percutánea, habitualmente se realizan con TC. La ecografía se encuentra subutilizada en el tórax debido a que posee limitaciones para el estudio del pulmón.

**Qué aporta este trabajo**

Este trabajo demuestra la experiencia de biopsias realizadas en forma percutánea, con ecografía en un hospital de alta complejidad. Se describen los resultados finales: eficacia y tasa de complicaciones y se las compara con reportes similares publicados en revistas internacionales.

**Divulgación**

La biopsia percutánea guiada por imágenes se ha convertido en una piedra angular en el tratamiento del cáncer. Habitualmente estas biopsias son llevadas a cabo con tomografía computada. La ecografía como guía de punción se encuentra subutilizada, pese a presentar varias ventajas sobre la TC como la ausencia de radiación, la visión en tiempo real de la aguja y del tumor y el bajo costo. Una de las desventajas de las biopsias con ecografía es la dependencia del operador. Este trabajo tiene como finalidad describir la experiencia en biopsias de imágenes pulmonares guiadas por ecografía en nuestro medio.

## Introducción

El cáncer de pulmón tiene la segunda incidencia anual de cánceres y es la principal causa de muerte vinculada al cáncer a nivel mundial. Su incidencia está en ascenso ^1^ . Por otro lado, el diagnóstico incidental de nódulos pulmonares cada vez es más frecuente, debido a la utilización creciente de la tomografía computada (TC) de tórax por otros motivos ^2^ .

Desde su concepción en 1976, la biopsia de imágenes pulmonares se ha convertido en un procedimiento central para el diagnóstico y tratamiento del cáncer pulmonar ^3^ . La toma de una muestra de tejido en forma percutánea permite su estudio mediante anatomía patológica, biología molecular o cultivo de distintos gérmenes ^4^ . Las biopsias de lesiones pulmonares en forma percutánea, al igual que las del mediastino y la pleura, tradicionalmente son llevadas a cabo con TC por su capacidad para demostrar con claridad los huesos y el pulmón aireado. Sin embargo, en ciertos casos, también pueden ser realizadas con ecografía. La ecografía se destaca dentro de los métodos guía por su capacidad para monitorear el procedimiento en tiempo real y la ausencia de radiación ionizante ^5-9^ . Otras ventajas de la ecografía son su bajo costo, portabilidad y disponibilidad. Además permite la detección de vasos sanguíneos en el trayecto de la punción mediante la función doppler ^9^ . Debido al tamaño de los ecógrafos, es posible realizar estudios ecográficos en la habitación del paciente internado evitando su traslado ^10^ . Se ha descrito que el uso de la ecografía pulmonar disminuye el uso del tomógrafo y consecuentemente la emisión de radiación ionizante ^11^ . Este punto es particularmente relevante al momento de estudiar población pediátrica ^12^ . Dentro de las limitaciones del método se encuentran la experiencia del operador y la ubicación del nódulo dentro del tórax^(8,13,14)^. Debido a que el pulmón aireado constituye una barrera para las ondas de ultrasonido, solamente aquellas lesiones en la pared torácica o en contacto con ella podrán ser biopsiadas con ecografía^(5,8-9)^.

El objetivo de este estudio es analizar en forma retrospectiva una cohorte de casos con y sin antecedentes oncológicos en estudio por lesiones pulmonares o pleurales evidenciadas por TC y biopsiadas con la guía de ecografía. Los resultados de su eficacia y complicaciones fueron analizados y contrastados con bibliografía internacional.

## Método

Estudio retrospectivo realizado en el Hospital Italiano de Buenos Aires, Argentina entre el mes de Enero del 2020 y Diciembre del 2021, durante la pandemia SARS-CoV-2.

Todos los pacientes presentaban lesiones pulmonares visibles por TC, algunos tenían antecedentes oncológicos.

Se incluyeron pacientes de ambos sexos, sin restricción etaria bajo atención ambulatoria e internación. Todos los pacientes recibieron consulta con un radiólogo intervencionista previo al procedimiento en la cual se explicó las características del mismo y las posibles complicaciones. Todos los procedimientos se realizaron con parámetros de coagulación normal (K.P.T.T., A.P.T.T. y recuento de plaquetas). Las lesiones a biopsiar se encontraban en la pared del tórax, espacio pleural o pulmón. Todas las lesiones pulmonares biopsiadas con ecografía se encontraban en contacto directo con la pleura visceral. La decisión de realizar los procedimientos bajo guía ecográfica fue multicausal, no obstante destacamos la intención de limitar la exposición a radiación, optimizar el uso del tomógrafo para fines diagnósticos no intervencionistas y minimizar los traslados de pacientes internados. Esta última situación fue motivada por el contexto epidemiológico global durante la cual se desarrolló la investigación. Los pacientes que no presentaban visibilidad de la lesión por obstrucción de un hueso o por interposición de aire fueron intervenidos mediante TC. Las complicaciones fueron catalogadas acorde a la clasificación de Clavien Dindo.

El estudio fue aprobado por el Comité de Ética de Protocolos de Investigación (CEPI) de la institución (protocolo N° 6592 PRIISA 8010).

### Análisis estadístico:

Se describieron las variables categóricas como frecuencias absolutas y relativas en porcentaje. Se presentaron las variables cuantitativas como media y desvío estándar o mediana e intervalo intercuartil según distribución observada.

Se compararon las variables continuas con T-test o test de Wilcoxon Rank, según distribución y las variables categóricas mediante chi2 o test exacto de Fischer según supuestos.

## Material

Todos los procedimientos fueron realizados por dos radiólogos intervencionistas con experiencia en biopsias. Los procedimientos de pacientes ambulatorios se realizaron en la sala de ecografía del sector de Radiología Intervencionista (Diagnóstico por Imágenes). Los procedimientos de los pacientes internados fueron realizados en su habitación. Se utilizaron dos ecógrafos acorde a disponibilidad: MyLab Twice (Esaote, Génova, Italia), Xario 200 y Nemio IStyle (Toshiba, Otawara, Japón). Se realizó ecografía del tórax con el paciente en posición supino, prono u oblicuo de acuerdo a la ubicación del nódulo. Se analizaron los espacios intercostales correspondientes con transductores lineal (7.5 MHz) o convexo (3.75Mhz) de acuerdo a la profundidad de visión requerida. Las biopsias se realizaron con técnica estéril, habiendo limpiado la piel del paciente con iodo-povidona y separado el área de punción con campos estériles. El transductor ecográfico fue cubierto con una funda estéril. Se colocó anestesia local (lidocaína 2% sin epinefrina) sobre la piel, las partes blandas y la pleura parietal mediante visión ecográfica. La punción se realizó con técnica de manos libres bajo apnea transitoria, con abordaje en plano (figura 1). Las biopsias fueron llevadas a cabo con agujas tipo Franseen (Argon Medical, Texas, USA) o con Kit para biopsia tipo Core (Bard Mission, Becton, Dickinson and Company, New Jersey, USA). Se emplearon agujas 18 o 20 gauge. En caso de desarrollar neumotórax, se realizaron maniobras de aspiración hasta lograr visualización del parénquima pulmonar nuevamente. Las muestras biológicas para estudio anatomopatológico fueron conservadas en formol al 10%, aquellos que requerían estudios complementarios de laboratorio fueron conservados en solución fisiológica y enviados a analizar inmediatamente. Todas ellas fueron revisadas por un citopatólogo al momento de la extracción, fijadas mediante etanol al 96%.

Al finalizar la punción, el paciente fue monitoreado durante dos horas en el área de observación. Se tomaron signos vitales, saturación de oxígeno y se realizó evaluación del dolor cada 15 minutos. Al cabo de las 2 horas se realizó control tomográfico para constatar ausencia de complicaciones y se le brindó el alta al paciente con indicaciones de reposo absoluto por 24 horas.

**Figura N° 1.**
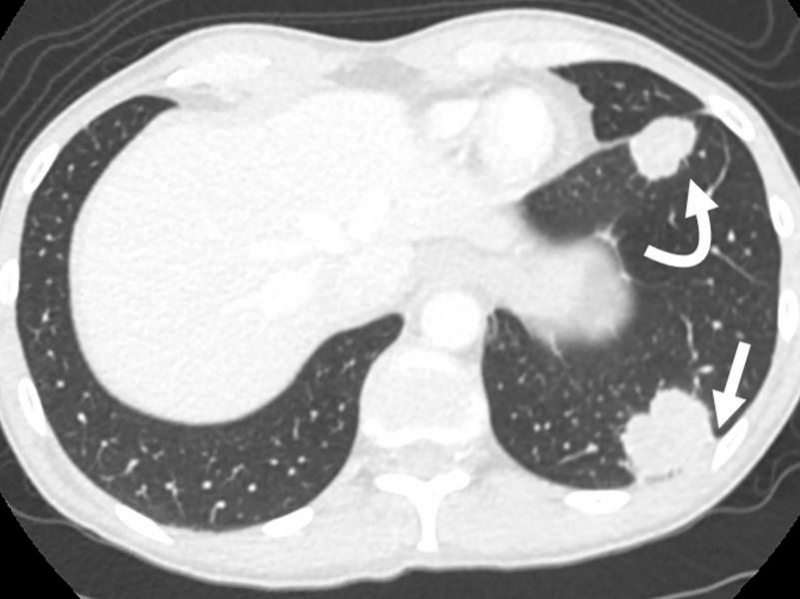
Mujer de 53 años con antecedentes de cáncer de cuello uterino en control imagenológico de rutina. Tomografía de tórax demuestra dos lesiones tumorales en el pulmón izquierdo, una periférica de base pleural (flecha) y otra profunda, rodeada de pulmón aireado (flecha curva).

**Figura N° 2:**
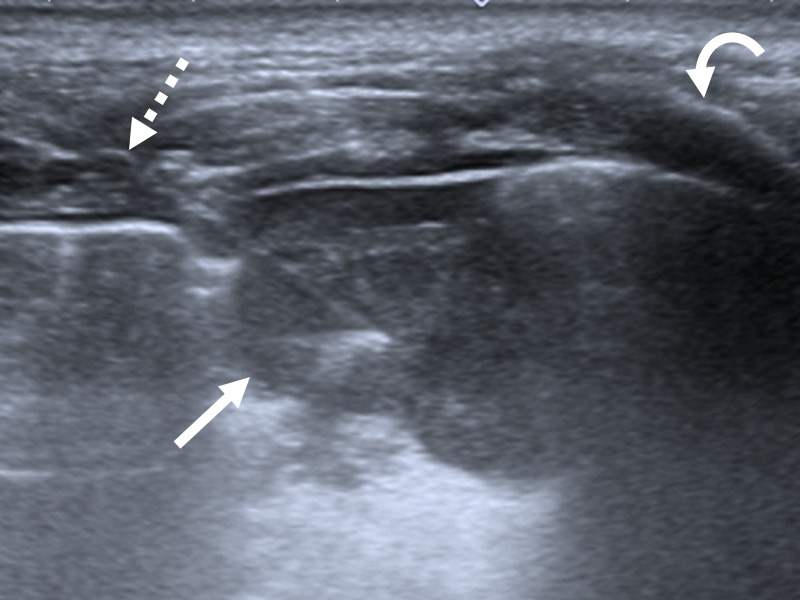
Biopsia percutánea de lesiones pulmonares expuestas en figura previa. Biopsia con ecografía demuestra lesión pulmonar periférica hipoecogénica (flecha) punzada con aguja tipo Franseen 18 gauge (flecha rayada). Nótese la costilla (flecha curva) como genera velamiento de la imagen subyacente a la misma.

## Resultados

Se realizaron 36 procedimientos. La mediana de edad fue de 73 años (IIQ 62-78,5). La mayor parte de las biopsias fueron de pulmón (29, el 80,6%) y las 7 restantes fueron de pleura. En 32 (88,9%) procedimientos la muestra obtenida presentó rédito diagnóstico y la prueba adicional más utilizada fue la inmunohistoquímica en 23 (63,9%) muestras, seguida por el cultivo y el FISH en 2 muestras respectivamente y se utilizó PCR en 1 muestra.

El rédito diagnóstico mostró que 19 (52,7%) lesiones eran tumores primarios de pulmón y pleura, 10 (27,7%) eran metástasis, 3 (8,3%) fueron tejido inflamatorio y en 4 (11,1%) no se obtuvo diagnóstico (tabla 1).

**Tabla N° 1:** Resumen de resultados, características de los pacientes y detalles de los procedimientos

Etiología	Edad	Comorbilidades	Localización de la lesión	Pruebas adicionales	Complicaciones	Superficie de contacto con la pleura (mm.)	Diámetro máximo abordable (mm.)	Aguja	Tipo de anestesia	Formato de atención
Patología inflamatoria	91	Síndrome confusional	Pleural	Cultivo	No	13	13	Franseen 18 gauge	Anestesia local	Internación
	74	Ninguna	Pulmón	Cultivo	No	37	29	Franseen 18 gauge	Anestesia local	Internación
	11	Ninguna	Pulmón	Ninguna	No	38	21	Franseen 18 gauge	Anestesia general	Ambulatoria
No diagnóstico	67	Síndrome confusional	Pulmón	Ninguna	Hemotórax moderado	128	59	Franseen 18 gauge	Anestesia local	Ambulatoria
	59	Ninguna	Pulmón	Ninguna	Neumotórax laminar	15	15	Franseen 18 gauge	Anestesia local	Ambulatoria
	70	Disnea clase funcional 2	Pulmón	Ninguna	No	17	64	Franseen 18 gauge	Anestesia local	Ambulatoria
	84	Síndrome confusional	Pulmón	Ninguna	No	67	58	Franseen 20 gauge	Anestesia local	Internación
Tumor primario	76	Ninguna	Pleural	Reacción en cadena de la polimerasa	No	59	40	Franseen 18 gauge	Anestesia local	Ambulatoria
	75	Ninguna	Pleural	Inmunohistoquímica	No	36,7	15	Franseen 18 gauge	Anestesia local	Ambulatoria
	62	Ninguna	Pleural	Inmunohistoquímica	No	50	24	Franseen 18 gauge	Anestesia local	Internación
	52	Ninguna	Pleural	Inmunohistoquímica	No	128	25	Corte 18 gauge	Anestesia local	Ambulatoria
	62	Ninguna	Pleural	Inmunohistoquímica	No	80	13	Corte 18 gauge	Anestesia local	Ambulatoria
	32	Ninguna	Pulmón	Hibridación fluorescente in situ	No	46	29	Franseen 18 gauge	Anestesia local	Internación
	77	E.P.O.C.	Pulmón	Inmunohistoquímica	No	17	15	Franseen 18 gauge	Anestesia local	Ambulatoria
	75	Ninguna	Pulmón	Inmunohistoquímica	No	43	54	Franseen 18 gauge	Anestesia local	Ambulatoria
	74	Disnea clase funcional 2	Pulmón	Inmunohistoquímica	No	23	27	Franseen 18 gauge	Anestesia local	Ambulatoria
	69	Ninguna	Pulmón	Inmunohistoquímica	No	15	12	Franseen 20 gauge	Anestesia local	Ambulatoria
	70	Ninguna	Pulmón	Inmunohistoquímica	No	64	54	Franseen 20 gauge	Anestesia local	Ambulatoria
	84	Ninguna	Pulmón	Inmunohistoquímica	No	73	32	Franseen 20 gauge	Anestesia local	Internación
	17	Ninguna	Pulmón	Hibridación fluorescente in situ	No	45	39	Corte 18 gauge	Anestesia general	Ambulatoria
	72	Ninguna	Pulmón	Ninguna	No	57	31	Corte 18 gauge	Anestesia local	Ambulatoria
	74	Hipoacusia	Pulmón	Ninguna	Neumotórax laminar	15	13	Corte 20 gauge	Anestesia local	Ambulatoria
	48	Disnea clase funcional 3	Pulmón	Inmunohistoquímica	No	50	55	Corte 20 gauge	Anestesia local	Ambulatoria
	86	Ninguna	Pulmón	Inmunohistoquímica	No	39	29	Corte 18 gauge	Anestesia local	Ambulatoria
	53	Ninguna	Pulmón	Inmunohistoquímica	No	19	31	Franseen 18 gauge	Anestesia local	Ambulatoria
	70	Ninguna	Pulmón	Inmunohistoquímica	No	35	36	Franseen 20 gauge	Anestesia local	Ambulatoria
Tumor secundario	79	Ninguna	Pleural	Inmunohistoquímica	No	45,9	35	Franseen 18 gauge	Anestesia local	Ambulatoria
	78	Ninguna	Pulmón	Inmunohistoquímica	Hemotórax leve	20	42	Franseen 18 gauge	Anestesia local	Ambulatoria
	69	Ninguna	Pulmón	Inmunohistoquímica	No	127	55	Franseen 18 gauge	Anestesia local	Ambulatoria
	78	Disnea clase funcional 2	Pulmón	Inmunohistoquímica	Dolor	20	20	Franseen 18 gauge	Anestesia local	Internación
	62	Ninguna	Pulmón	Inmunohistoquímica	No	18	18	Franseen 18 gauge	Anestesia local	Ambulatoria
	89	Ninguna	Pulmón	Inmunohistoquímica	No	29	13	Franseen 18 gauge	Anestesia local	Internación
	66	Disnea clase funcional 3	Pulmón	Inmunohistoquímica	No	71	33	Franseen 18 gauge	Anestesia local	Internación
	81	Ninguna	Pulmón	Inmunohistoquímica	No	36	49	Franseen 18 gauge	Anestesia local	Ambulatoria
	83	Ninguna	Pulmón	Inmunohistoquímica	No	20	31	Franseen 18 gauge	Anestesia local	Ambulatoria
	80	Ninguna	Pulmón	Ninguna	No	25	29	Corte 18 gauge	Anestesia local	Ambulatoria

El diámetro máximo abordable de la lesión (en mm), previo a transgredir pulmón sano fue de 30 (19-41).

La mediana de superficie en contacto con la pleura, en mm, fue de 37,5 (IIQ 20-58).

Se halló que las lesiones de mayor diámetro abordable tuvieron menor rédito diagnóstico (p=0,01) pero no se halló asociación entre el diámetro en contacto con la pleura y el rédito diagnóstico (p=0,43).

Se reportaron complicaciones en 5 (13,9%) pacientes: 2 con neumotórax leve, 2 con hemotórax (1 leve y 1 moderado) y 1 paciente refirió dolor.

La aguja más utilizada fue la Franseen 18G en 23 procedimientos, seguida por aguja de corte semiautomática 18G en 6, la Franseen 20G en 5 procedimientos y finalmente la aguja de corte semiautomática 20G en 2.

Del total, 27 procedimientos se realizaron por ambulatorio y 9 se realizaron en pacientes internados, 34 se realizaron con anestesia local y sólo 2 requirieron anestesia general (población pediátrica).

## Discusión

En este estudio se describe una cohorte de pacientes con lesiones pulmonares y pleurales, a los cuales se le realizó una biopsia bajo guía ecográfica. La eficacia diagnóstica y la tasa de complicaciones han demostrado estar en línea con la experiencia de otros autores y guías internacionales.

Heerink y col. realizaron un metaanálisis, para valoración de complicaciones en biopsias pulmonares en 4620 procedimientos realizados con aguja fina y 8133 procedimientos con aguja gruesa. Allí encontraron la presencia de complicaciones en el 24% y 38% de los procedimientos aunque las complicaciones mayores fueron descritas en el orden del 4.4 y 5.7% respectivamente. La complicación más frecuente fue el neumotórax en el 25.3% de los casos intervenidos con aguja gruesa pero solo el 5.6% requirió colocación de drenaje. Adicionalmente la presencia de hemotórax solo fue mencionada en algunos artículos (6/46) y su análisis no fue considerado para el metaanálisis. Debemos mencionar que solamente se incluyeron biopsias de lesiones pulmonares intervenidas con guía tomográfica pero no hacen mención a la posición de los tumores respecto a la pleura ni su profundidad ^15^ . Hay que destacar que la incidencia del neumotórax en este tipo de práctica aumenta con la profundidad de la lesión respecto a la pleura y el grosor de la aguja ^16^ . Entonces, es de esperar que los procedimientos efectuados con ecografía, donde solamente se pueden intervenir lesiones con contacto pleural, la incidencia de neumotórax sea menor ^9, 16^ . Tal es así que Lemieux y col., en 528 biopsias de lesiones pulmonares guiadas por ecografía, encontraron neumotórax en el 15% de los casos estudiados ^17^ .

Para profundizar, Sconfienza y col. compararon biopsias de lesiones pulmonares periféricas en contacto pleural mediante TC y ecográfica, realizados con aguja 18 gauge, y encontraron presencia de neumotórax en el 11.4% y solamente 3 casos requirieron drenaje posterior (datos coleccionados). Precisamente, en los procedimientos llevados a cabo con ecografía se evidenció neumotórax en el 5.8% (6/103) mientras que con TC se lo observó en el 14.7% (25/170) ^18^ . Análogamente Lee y col. encontraron 7% de complicaciones en procedimientos guiados por ecografía vs. 24% en TC. Del total de procedimientos guiados por ecografía (150) encontraron 10 neumotórax, solo uno requirió drenaje y el resto se trató de complicaciones auto-limitadas ^19^ .

En nuestra experiencia solamente encontramos 5 complicaciones (13.9%) y todas ellas leves, sin requerimiento de procedimientos adicionales. Lo evidenciado aquí se encuentra por debajo de las encontradas por Heenrik. Por ejemplo las guías para implementación de mejoras de la Sociedad de Radiología Intervencionista (S.I.R.) mencionan posibilidad de neumotórax en el orden del 15,3-22%, sin embargo dicha publicación no hace referencia al método guía ni a la relación de los tumores con la pleura ^20^ . Para biopsias de lesiones periféricas, el riesgo de neumotórax disminuye cuanto mayor sea el área de contacto entre la lesión y la pleura. Inversamente, el rédito diagnóstico aumenta al aumentar la superficie de contacto entre ambas ^17^ . Tal es así, que de los pacientes que sufrieron neumotórax, las lesiones intervenidas tenían una superficie de contacto pleural de 15mm, las menores en este trabajo. Nosotros constatamos que la tasa de neumotórax fue de aproximadamente el 5.5% y en ningún caso se requirió colocación de tubo de drenaje.

Se ha estipulado que el rédito diagnóstico para las biopsias de lesiones pulmonares es del 92.6-93.4% dependiendo del tamaño de la lesión y se ha recomendado superar una tasa de éxito del 80% para ser considerado eficaz en la práctica diaria. Pese a esta declaración, las guías del S.I.R. hacen hincapié en que no es conveniente generalizar ya que dependiendo de la ubicación de la lesión y aguja utilizada estos porcentajes pueden variar. Con respecto a estudios complementarios sobre el material biológico obtenido, la tasa de éxito para estudio mediante secuenciación de próxima generación es del 93.1 ± 5.4% ^20^ . Otros autores, comparando entre ambos métodos guía, han remarcado que el éxito de las biopsias con ecografía fue de 93.4% vs. 84.3% con TC. Esto fue atribuido a que con ecografía es "más seguro" repetir la toma de muestra. También hicieron hincapié en que lesiones de mayor tamaño tienden a tener resultados no diagnósticos, probablemente por presencia de necrosis ^21^ . La eficacia diagnóstica en ésta experiencia fue de 88.9%% (4 resultados fueron no diagnósticos). Aquí no hemos medido el diámetro de las lesiones intervenidas ya que muchas de ellas presentaban forma irregular. Sin embargo hemos medido el diámetro máximo abordable, o sea la cantidad de milímetros pasibles de ser biopsiados de acuerdo a la incidencia de la aguja. Adicionalmente, tuvimos en cuenta la cantidad de milímetros entre los cuales la lesión contactaba la pleura. Llama la atención que las lesiones que presentaban mayor volumen se asociaron a menor rédito diagnóstico: hallazgo relacionado a la presencia de necrosis. Por el contrario, el área de contacto entre la lesión y la pleura no se asoció a variaciones en el diagnóstico. La geometría de las lesiones pudo haber sido determinante, lesiones con amplio contacto pleural podrían presentar poca profundidad o viceversa. En este último caso, en lesiones con contacto pleural limitado pero gran profundidad dada la forma de abordaje con ecografía, la TC podría ser de mayor utilidad. Dicha elección creemos que depende de la experiencia del operador. Un estudio, con mayor cantidad de pacientes, relacionando el tamaño de la lesión (en milímetros abordables acorde a la dirección de la aguja) y la superficie de contacto podría ser necesario para aclarar esta situación.

Los costos estimados por procedimiento también difieren. Sconfienza y col. mencionan que los procedimientos guiados por ecografía equivalen al 56.8-67.5% del total de aquellos guiados por TC ^18^ .

Aunque aquí no realizamos comentarios sobre variables no cuantificadas, por ejemplo duración del procedimiento, otros autores han mencionado que las biopsias guiadas por ecografía son más ágiles y resultan en menor tiempo de intervención que aquellas guiadas por TC. Diferentes comentarios han sido realizados al respecto por ejemplo la habilidad del operador y la visión en tiempo real en defensa de esta observación ^19^ .

Este trabajo presenta algunas debilidades que merecen atención. Se trata de una cohorte retrospectiva y la cantidad de casos es limitada. Más aún, las intervenciones fueron llevadas a cabo por dos operadores con amplia experiencia en biopsias guiadas por imágenes, y probablemente esto no pueda ser replicado en otras instituciones. Otro punto a mencionar es que aquí no comparamos los resultados obtenidos con otra metodología guía. Pese a esto y habiendo remarcado las virtudes de la ecografía, creemos que éste trabajo debe servir como incentivo para realizar biopsias de lesiones pulmonares en contacto pleural con ecografía cada vez que sea posible. Dicho cambio disminuiría la exposición a radiación ionizante del paciente en estudio. Más aún, se logra reducir costos de la práctica y permite que nuestra población tenga mayor acceso a biopsias percutáneas.

## Conclusión

Nuestra experiencia, al igual que la de otros autores, ha demostrado que la ecografía es un método válido para ser usado como guía de biopsias de lesiones pleurales y pulmonares periféricas. La tasa de complicaciones y reedito diagnóstico ha demostrado estar en línea con la experiencia de otros autores y guías internacionales. Éste trabajo abre la posibilidad de re-direccionar pacientes seleccionados, que se hubiesen intervenido con TC, a ecografía y poder así utilizar las virtudes del método.
